# Supplementary material for: PD-L1 Expression in Cutaneous Angiosarcomas: A Systematic Review with Meta-Analysis
Source: Curr Oncol. 2023 May 17;30(5):5135–44. doi: 10.3390/curroncol30050388 (PMC10217767; doi:10.3390/curroncol30050388)
Supplement: Supplementary file 1 [file curroncol-30-00388-s001.zip › Supplementary Table 1.pdf]

Supplementary Table 1. The Joanna Briggs Institute critical appraisal checklist.

Study	Was the sample frame appropriate to address the target population?	Were study participants sampled in an appropriate way?	Was the sample size adequate?	Were the study subjects and the setting described in detail?	Was the data analysis conducted with sufficient coverage of the identified sample?	Were valid methods used for the identification of the condition?	Was the condition measured in a standard, reliable way for all participants?	Was there appropriate statistical analysis?	Was the response rate adequate, and if not, was the low response rate managed appropriately?	Risk of Bias
Shimizu et al.	Yes	Yes	Yes	Yes	Yes	Yes	Yes	Yes	Yes	Low
Botti et al.	No	Yes	No	Yes	Yes	Yes	Yes	No	Yes	High
Honda et al.	Yes	Yes	Yes	Yes	Yes	Yes	Yes	Yes	Yes	Low
Kawamura et al.	Yes	Yes	Yes	Yes	Yes	Yes	Yes	No	Yes	Low
Gambichler et al.	Yes	Yes	Yes	Yes	Yes	Yes	Yes	Yes	Yes	Low
Okabayshi et al.	Yes	Yes	Yes	Yes	Yes	Yes	Yes	Yes	Yes	Low
Bi et al.	Yes	Yes	Yes	Yes	Yes	Yes	Yes	Yes	Yes	Low
Espejo-Freire et al.	No	Yes	No	Yes	Yes	Yes	Yes	No	Yes	High
Googe et al.	No	Yes	No	Yes	Yes	Yes	Yes	No	Yes	High
Tomassen et al.	Yes	Yes	Yes	Yes	Yes	Yes	Yes	Yes	Yes	Low
